# Exercise Training Induced Cardioprotection with Moderate Hyperglycemia versus Sedentary Intensive Glycemic Control in Type 1 Diabetic Rats

**DOI:** 10.1155/2018/8485624

**Published:** 2018-07-05

**Authors:** Matthew W. McDonald, Michelle S. Dotzert, Mao Jiang, Michael R. Murray, Earl G. Noble, C. W. James Melling

**Affiliations:** ^1^Exercise Biochemistry Laboratory, School of Kinesiology, Western University, London, ON, Canada; ^2^Lawson Health Research Institute, London, ON, Canada

## Abstract

Intensive insulin therapy (IIT; 4–7 mmol/L) is the preferred treatment for type 1 diabetes mellitus (T1DM) patients to reduce the risk of cardiovascular disease (CVD). However, this treatment strategy has been questioned as it is accompanied with a sedentary lifestyle leading to weight gain and insulin resistance. T1DM patients who partake in high-intensity aerobic training (AT_high_) to reduce CVD often utilize conventional insulin therapy (CIT; 9–15 mmol/L) to offset the risk of hypoglycemia. Moreover, exercise modalities incorporating resistance training (RT) have been shown to further reduce this risk. The purpose of this investigation was twofold: (1) to determine if CIT paired with AT_high_ results in larger cardioprotection from an ischemia-reperfusion (I-R) injury than IIT and (2) to establish if the integration of RT with AT_high_ (ART) results in similar cardioprotection as AT_high_. Diabetic (D) male Sprague-Dawley rats were divided into D-IIT (*n* = 12), D-CIT (*n* = 12), D-AT_high_ (*n* = 8), D-RT (*n* = 8), and D-ART (*n* = 8). T1DM was induced with streptozotocin, and blood glucose was adjusted with insulin. D-AT_high_ occurred on a treadmill (27 m/min; 1 hr), D-RT performed weighted ladder climbs, and D-ART alternated daily between AT_high_ and RT. Exercise occurred 5 days/wk for 12 wks. This investigation demonstrates that cardioprotection following an I-R injury was similar between D-AT_high_ and D-IIT. This cardioprotection is not exercise-specific, and each provides unique advantages. D-AT_high_ leads to improved glycemia while insulin sensitivity was enhanced following resistance exercises. Thus, exercise is an effective means to elicit cardioprotection in T1DM. However, in addition to glycemia, other factors should be considered when tailoring an exercise program for T1DM patients.

## 1. Introduction

Individuals with type 1 diabetes mellitus (T1DM) exhibit a heightened risk for cardiovascular disease (CVD) not entirely accounted for by traditional risk factors (hyperglycemia, obesity, hypertension, dyslipidemia, and smoking) [1]. To date, the most characterized strategies to limit CVD development have been intensive insulin therapy (IIT) [2] and regular exercise [3–5]. However, both IIT and exercise potentiate the risk of hypoglycemia, especially when attempted collectively [2, 6]. To counteract hypoglycemia risk, individuals with T1DM often intentionally elevate their blood glucose concentrations prior to exercise through changes in insulin dosing and/or carbohydrate ingestion [7]. As such, individuals with T1DM who are more physically active typically prescribe to a more conventional insulin therapy (CIT) and have higher HbA1c values with reduced focus on glycemic control [8].

Additional work is needed to better evaluate the cardiovascular benefits and risks associated with regular exercise in physically active individuals with T1DM that often prescribe to less stringent glycemic control, since elevations in glycemia (HbA_1c_) are known to increase the risk of cardiovascular complications [2]. Our group has demonstrated that the combination of less stringent blood glucose control and high-intensity aerobic exercise training (AT_high_) in experimental T1DM rats not only decreases the risk of exercise-induced hypoglycemia [4] but also has numerous cardiovascular benefits such as increased recovery from an ischemic insult [4], reduction in cardiovascular autonomic dysfunction [9], improvement in systolic and diastolic heart function [5], and improved vascular reactivity [10, 11]. It is unknown how these cardiovascular benefits would compare to stringent blood glucose control alone (i.e., IIT), the predominant treatment option for individuals with T1DM [2, 12]. This is a significant question that needs to be answered since IIT is associated with cardiovascular risk factors such as increased sedentary behaviour, weight gain, and insulin resistance [8, 13, 14].

Additionally, it has been established both experimentally [15] and clinically [16] that poor glycemic control leads to hepatic glycogen deficiencies. Restoration of hepatic glycogen content could represent a mechanism for combatting hypoglycemia, as hepatic glycogen is the predominant source of blood glucose during exercise [17] and insulin overcorrection [18]. Our laboratory has recently shown that ten weeks of AT_high_ fails to normalize hepatic glycogen in T1DM rats despite significantly elevated levels of hepatic glycogenic storage enzymes [15]. In contrast, resistance training (RT) has been shown to increase hepatic glycogen content in rats [19], while also alleviating the risk of exercise-induced hypoglycemia in T1DM [4, 20]. While still allowing for the cardiovascular benefits associated with regular aerobic exercise, the integration of RT with aerobic exercise may allow individuals with T1DM to exercise safely by reducing the risk of hypoglycemia development. Indeed, the Canadian Diabetes Association recommends that RT be incorporated into aerobic exercise regimes at least twice a week [21].

The objective of the present study was to examine whether moderate blood glucose control and AT_high_ result in greater levels of cardioprotection than more stringent blood glucose control. Secondly, it was determined whether combining RT with AT_high_ resulted in similar cardioprotection and less exercise-induced blood glucose fluctuations. Additionally, the potential relationship between glycemic status and cardioprotection was explored.

## 2. Methods

This study was approved by the Research Ethics Board of the University of Western Ontario which is in compliance with the guidelines of the Canadian Council on Animal Care. Eight-week-old male Sprague-Dawley rats were obtained from Charles River Laboratories, provided standard rat chow ad libitum, and housed in pairs at a standard temperature and humidity (21.5°C and 50% humidity).

### 2.1. Experimental Protocol

Sprague-Dawley rats were randomly divided into one of five diabetic groups (D): conventional insulin therapy (D-CIT; *n* = 12), intensive insulin therapy (D-IIT; *n* = 12), high-intensity aerobic exercise training (D-AT_high_; *n* = 8), resistance exercise training (D-RT; *n* = 8), and combination aerobic/resistance exercise training (D-ART; *n* = 8). During experimental week one, T1DM was induced after five consecutive daily injections of streptozotocin (Sigma-Aldrich; 20 mg/kg; dissolved in 0.1 M citrate buffer, pH 4.5) and T1DM was confirmed after two nonfasting blood glucose concentrations greater than 18 mmol/L. After diabetes confirmation, subcutaneous insulin pellets (Linshin, Toronto, Canada) were implanted in the abdomen (experimental week two). Through insulin pellet adjustments, it was intended to maintain blood glucose concentrations in D-CIT, D-AT_high_, D-RT, and D-ART between 9 and 15 mmol/L and D-IIT between 4 and 9 mmol/L. Exercise training occurred five times a week over a twelve-week period (experimental week 3 to 14). D-AT_high_ rats exercised on a motorized treadmill at 27 m/min (six percent grade) for one hour. Continuous running was encouraged by small blasts of compressed air at the rear of the treadmill. In D-RT rats, resistance training consisted of climbing a vertical ladder with weights secured to the proximal portion of the tail, as previously described [4]. Familiarization occurred the week prior to training (experimental week 2) and consisted of 10 climbs a day with varying weights attached (5%, 15%, 20%, and 35% of each rat's body mass). Regular resistance training sessions (experimental week 3 to 14) consisted of incremental increases in weight (50%, 75%, and 90% of maximal lifting capacity) followed by 100% of their maximal lifting capacity until exhaustion (unable to finish climb despite tactile stimulation to haunches). Maximum lifting capacity was calculated every fourth exercise session and was determined by sequentially adding 30 grams of weight to the rat's tail until exhaustion (starting at 75% of their body mass). In D-ART rats, exercise training consisted of alternating daily between the aerobic and resistance exercises.

### 2.2. Blood Analysis

Blood samples were taken over two consecutive days from the saphenous vein during the last week of exercise training (experimental week 14; pre/postexercise) to determine if antecedent AT_high_ or RT altered the blood glucose response to a subsequent exercise bout [22]. In D-ART, this measure was conducted at week 11 and week 12 of training (experimental week 13 and 14, resp.) to determine if performing AT_high_ (or RT) first had an effect on glucoregulation following a subsequent bout of RT (or AT_high_). Blood glucose concentrations were detected using a OneTouch Ultra 2 Blood Glucose Monitoring System (Lifescan Canada Ltd., Burnaby, BC, Canada) and OneTouch test strips (Lifescan Canada Ltd.). Epinephrine concentrations prior to and after exercise were determined via ELISA (Cusabio, catalog number CSB-E08678r). Fructosamine concentrations were determined using the procedure outlined by Oppel et al. [23]. Briefly, serum samples taken at the completion of the study were added to a carbonate buffer (pH 10.8) containing 0.25 mM nitroblue tetrazolium (NBT) at 37°C. Following a 20-minute incubation at 37°C, the reaction was read at 530 nm and compared to standards of 1-deoxy,1-morpholinofructose (DMF; Sigma-Aldrich) and albumin (40 g/L).

### 2.3. Langendorf Heart Preparation

Three days following the last exercise bout, all rats were anaesthetized with isoflurane and hearts were extracted and placed in cold Krebs-Henseleit buffer (KHB; 120 mM NaCl, 4.63 mM KCl, 1.17 mM KH_2_PO_4_, 1.25 mM CaCl_2_, 1.2 mM MgCl_2_, 20 mM NaHCO_3_, and 8 mmol/L glucose). Hearts were rapidly cannulated for unpaced retrograde perfusion of KHB (37°C; gassed with 95% O_2_ and 5% CO_2_) at 15 mL/min. A small water-filled latex balloon was inserted through the mitral valve and into the left ventricle. Hearts were equilibrated to the preparation for 30 minutes (preischemia) followed by the termination of flow for 50 minutes. Subsequently, reperfusion occurred for a total of 30 minutes at 15 m/min. Left ventricle pressures (LVDP, left ventricle developed pressure; LVEDP, left ventricle end-diastolic pressure) were measured with a pressure transducer (Statham Gould P23ID), and the rate of pressure development (+dp/dt) and relaxation (−dp/dt) were obtained using a PowerLab 8/30 data acquisition system and analyzed by LabChart 7.0 Pro software (ADInstruments, Colorado Springs, Colorado, USA). Area under the curve (AUC) was determined for the pressure curves of each rat in the study in order to correlate measures to glycemic control and insulin resistance.

### 2.4. Glucose Tolerance Test

Intravenous glucose tolerance tests (IVGTT) were conducted following training (experimental week 14) after an 8–12-hour fast and consisted of a sterile injection (1 g/kg) of dextrose solution (50% dextrose, 50% ddH_2_O) into the lateral tail vein. Blood glucose concentrations were measured at 5, 10, 20, 30, and 40 minutes postinjection, and area under the curves (AUC) were determined for each individual rat. Prior to the IVGTT, blood samples were taken from the saphenous vein and exogenous insulin concentrations were measured via ELISA (Alpco, Salem, NH: catalog number 80-INSHU-E01.1). The measure of insulin resistance was considered the AUC of the IVGTT multiplied by exogenous insulin concentration. We have previously reported that when using this T1DM model, sedentary rats can become insulin resistant and require substantial more exogenous insulin in order to maintain the desired blood glucose concentrations [15, 24]. Accordingly, when determining the insulin resistance measure, the amount of circulating insulin present in the rat during the IVGTT was factored into the calculation.

### 2.5. Western Blotting

Liver (extracted during sacrifice at end of study) and left ventricles were homogenized in buffer (100 mM NaCl, 50 mM Tris base, 0.1 mM EDTA, and 0.1 EGTA, pH ~7.5) using a polytron, and total protein concentrations were determined by the Bradford protein assay. Homogenates (40–80 *μ*g of protein) were mixed with equal volumes of sample buffer (0.125 M Tris, 20% glycerol, 4% SDS, 10% *β*-mercaptoethanol, 0.015% bromophenol blue, pH ~6.8), separated by SDS-PAGE (4% stacking, 10% separating) and transferred to nitrocellulose membranes. Membranes were blocked in 5% nonfat dairy milk in TTBS (10 mM Tris, 100 mM NaCl, and 0.1% Tween-20, pH 7.5) for 1 hour and incubated overnight at 4°C with primary antibodies (Cell Signaling: Hsp70 1 : 4000, glycogen synthase 1 : 1000; Abcam: glycogen phosphorylase 1 : 2000, SERCA2 1 : 1000; Santa Cruz: glucose-6-phosphotase 1 : 200) diluted in TTBS with 2% nonfat dairy milk. Following washes in TTBS, membranes were exposed to corresponding secondary antibodies (IgG-HRP conjugated, Bio-Rad) in TTBS with 2% nonfat dairy milk for 1 hour at room temperature. After successive washes in TTBS, protein bands were visualized with a luminol-based chemiluminescent substrate (Western C Enhanced Chemiluminescent Kit; Bio-Rad), imaged with the Chemidoc XRS System (Bio-rad), and analyzed with Quantity One Software (Bio-Rad). Optical densities were normalized to a consistent non-T1DM control sample and subsequently *β*-actin.

### 2.6. Statistical Analysis

Body mass, blood glucose, fructosamine, exogenous insulin, insulin resistance, and Western blot data were compared using a one-way analysis of variance (ANOVA). Langendorf measures were compared using a two-way repeated measure ANOVA. Blood glucose concentrations and epinephrine concentrations in response to exercise, and over consecutive days, were compared using a two-way repeated measures ANOVA. When a significant difference was found, a least squares difference post hoc test was performed and significance was set at *p* < 0.05. Relationships between left ventricular mechanical performance and fructosamine or insulin resistance were determined via Pearson correlation. All data are presented as a mean ± standard error. All statistical analyses were completed using GraphPad Prism 6.

## 3. Results

### 3.1. Animal Characteristics

Blood glucose concentrations were lower in D-IIT compared to D-CIT (*p*=0.03), D-AT_high_ (*p* = 0.02), and D-ART (*p* = 0.007), and lower in D-RT compared to D-ART (*p* = 0.04) (Table 1). Body mass was higher in D-IIT compared to D-AT_high_ (*p* = 0.003), D-RT (*p* = 0.01), and D-ART (*p* < 0.04). Fructosamine concentrations were lower in D-IIT compared to D-CIT (*p* < 0.001) and D-ART (*p* = 0.01). Further, fructosamine concentrations were lower in D-AT_high_ compared to D-CIT (*p* = 0.003) and D-ART (*p* = 0.04). Exogenous insulin concentrations were lower in D-RT compared to D-IIT (*p* = 0.01), and lower in D-ART compared to D-CIT (*p* = 0.02) and D-IIT (*p* < 0.001). The insulin resistance measure was higher in D-IIT compared to D-AT_high_ (*p* = 0.02), D-RT (*p* = 0.008), and D-ART (*p* = 0.003). Further, D-CIT displayed higher insulin resistance compared to D-ART (*p* = 0.04).

### 3.2. Left Ventricular Mechanical Performance

**Figure 1:**
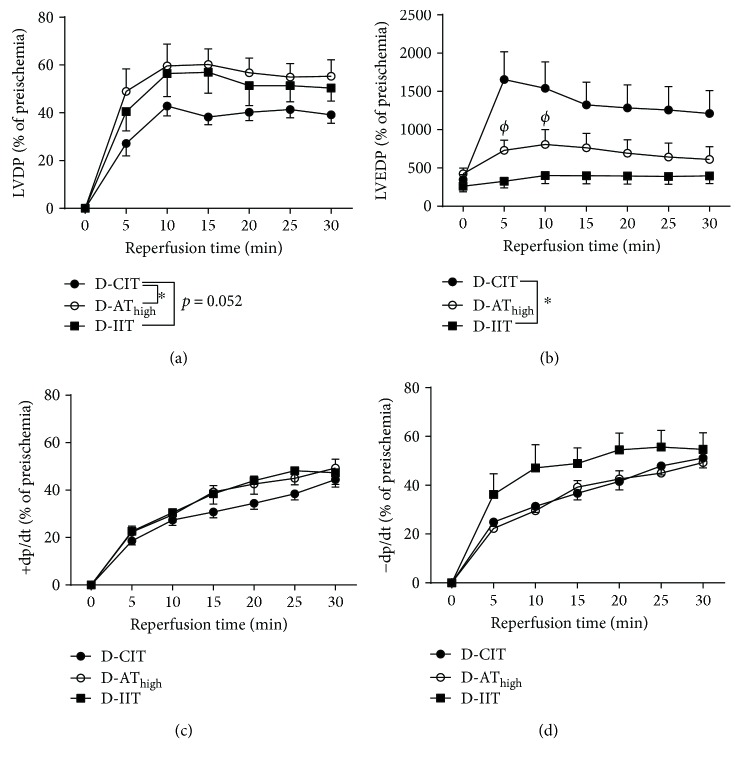
Left ventricle mechanical performance during ischemia-reperfusion. The data are presented in time course format. LVDP (a), LVEDP (b), +dP/dt (c), −dP/dt (d). ^∗^Significant main effect (*p* < 0.05); (φ) different from D-CIT (*p* < 0.05). Data are presented as a mean ± SE.

For the first objective of the study, we compared left ventricular mechanical performance following ischemia in D-CIT, D-AT_high_, and D-IIT. There was a significant increase in LVDP in D-AT_high_ compared to D-CIT (Figure 1(a); *p* = 0.03). No difference in LVDP was observed between D-AT_high_ and D-IIT (*p* = 0.5), and LVDP between D-CIT and D-IIT did not reach significance (*p* = 0.052). LVEDP was lower in D-IIT compared to D-CIT (Figure 4.1B; *p* = 0.004), while D-AT_high_ was lower than D-CIT at 5 (*p* < 0.0001) and 10 minutes (*p* = 0.03) during reperfusion. There was no difference in +dp/dt (*p* = 0.4) or –dp/dt (*p* = 0.2) across any of the groups (Figures 1(c) and 1(d)).

**Figure 2:**
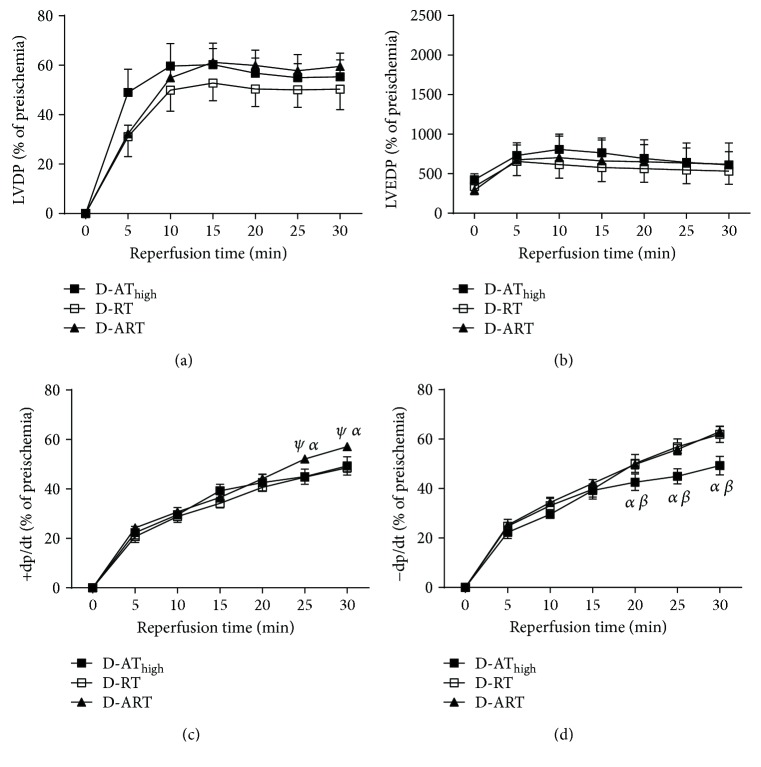
Left ventricle mechanical performance during ischemia-reperfusion and following different modalities of exercise training. The data are presented in time course format. LVDP (a), LVEDP (b), +dP/dt (c), −dP/dt (d). *ψ*: different from D‐AT_high_ (*p* < 0.05); *α*: different from D-RT (*p* < 0.05); *β*: different from D-ART (*p* < 0.05). Data are presented as a mean ± SE.

For the second objective of the study, we compared left ventricle mechanical performance following ischemia in D-AT_high_, D-RT, and D-ART. There were no differences in LVDP (*p* = 0.6) or LVEDP (*p* = 0.9) among D-AT_high_, D-RT, and D-ART (Figures 2(a) and 2(b)). Compared to D-AT_high_, D-ART had a higher +dp/dt at 25 (*p* = 0.01) and 30 minutes (*p* = 0.005) during reperfusion (Figure 2(c)). Compared to D-RT, D-ART had a higher +dp/dt at 25 (*p* = 0.009) and 30 minutes (*p* = 0.002) during reperfusion. D-AT_high_ had a slower –dp/dt than D-RT at 20 (*p* = 0.04), 25 (*p* = 0.001), and 30 minutes (*p* < 0.0001) (Figure 2(d)). Further, D-AT_high_ had a slower –dp/dt than D-ART at 20 (*p* = 0.04), 25 (*p* = 0.002), and 30 minutes (*p* < 0.0001).

### 3.3. Correlations of Left Ventricular Mechanical Performance

There was a significant correlation between the AUC of LVDP and fructosamine concentration (Table 2; *p* = 0.01; *r* = −0.4), while no correlation was evident between the AUC of LVEDP (*p* = 0.8), +dp/dt (*p* = 0.7), −dp/dt (*p* = 0.8), and fructosamine concentration. There was a significant correlation between the AUC of +dp/dt and insulin resistance (*p* = 0.03; *r* = −0.4), but no correlation between insulin resistance and the AUC of LVDP (*p* = 0.7), LVEDP (*p* = 0.65), or –dp/dt (*p* = 0.5).

### 3.4. Molecular Analysis

**Figure 3:**
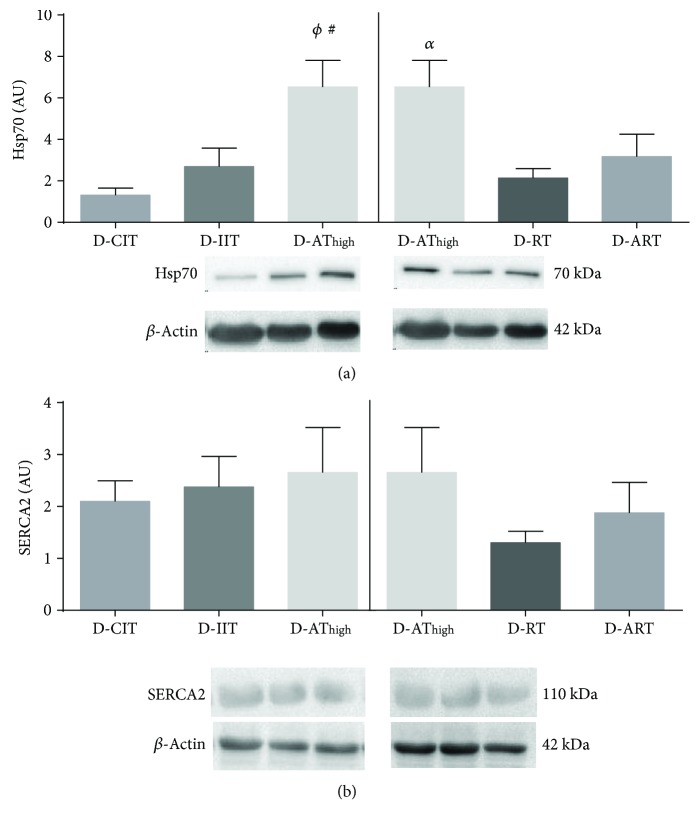
Left ventricle Hsp70 (a) and SERCA2 (b) protein content. *ϕ*: different from D-CIT; #: different from D-IIT; *α*: different from D-RT. Significance *p* < 0.05. Data are presented as a mean ± SE.

An elevation in left ventricle Hsp70 content was evident in D-AT_high_ compared to both D-CIT (*p* = 0.003) and D-IIT (*p* = 0.009) (Figure 3(a)), while no differences were evident in SERCA2 among D-CIT, D-IIT, and D-AT_high_ (Figure 3(b); *p* = 0.8). Differences existed between exercise regimes in that D-AT_high_ resulted in higher left ventricle Hsp70 compared to D-RT (*p* = 0.02), but did not differ significantly from D-ART (Figure 3(a); *p* = 0.1). No differences in SERCA2 expression were evident among exercise regimes (Figure 4.3B; *p* = 0.4).

### 3.5. Hepatic Glycogen Content and Regulatory Enzymes

**Figure 4:**
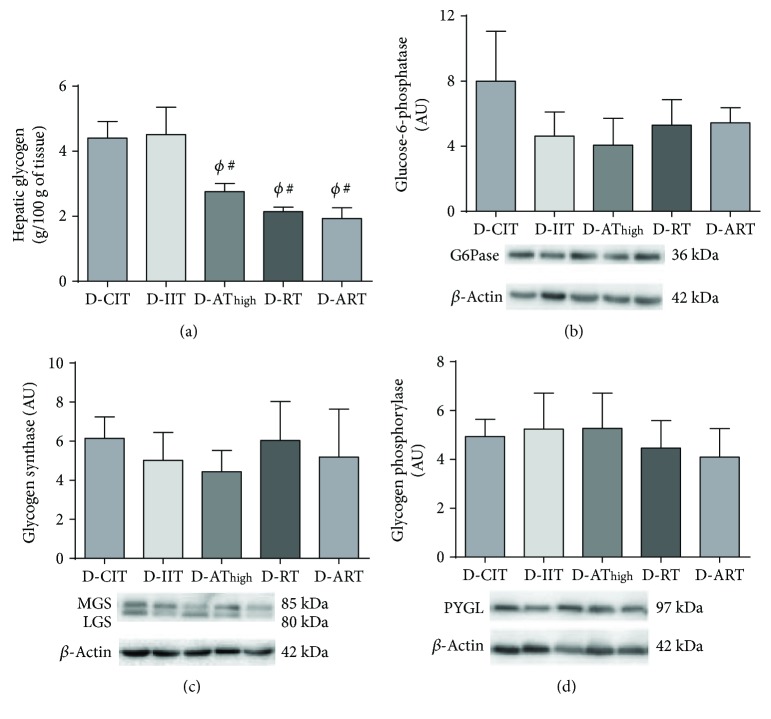
Hepatic glycogen content (a), glycogen-6-phosphatase (b), glycogen synthase (MGS: muscle glycogen synthase; LGS: liver glycogen synthase) (c), and glycogen phosphorylase (d). *ϕ*: different from D-CIT (*p* < 0.05); #: different from D-IIT (*p* < 0.05). Data are presented as a mean ± SE.

Hepatic glycogen content was higher in D-CIT compared to D-AT_high_ (*p* = 0.05), D-RT (*p* = 0.01), and D-ART (*p* = 0.004) (Figure 4(a)). Hepatic glycogen was also higher in D-IIT compared to D-AT_high_ (*p* = 0.04), D-RT (*p* = 0.01), and D-ART (*p* = 0.004). No differences in glycogen synthase (*p* = 0.9), glycogen phosphorylase (*p* = 0.9), and glycogen-6-phosphatase (*p* = 0.7) were apparent between experimental groups (Figures 4(b)–4(d)).

### 3.6. Exercise-Mediated Changes in Blood Glucose

Significant declines in blood glucose concentrations following exercise were apparent in D-AT_high_ at day 1 and day 2 of exercise (week 12 of training; Table 3; *p* < 0.0001). No change in blood glucose concentrations was apparent following RT at day 1 or day 2 (week 12 of training; *p* = 0.5). In D-ART, significant declines in blood glucose concentrations were apparent only on AT_high_ days at both week 11 (*p* = 0.0003) and week 12 (*p* < 0.0001) of training. In D-AT_high_, no change in epinephrine concentrations was evident from pre to postexercise (week 12 of training; *p* = 0.4; Table 4), and epinephrine concentrations were similar between day 1 and day 2 (week 12 of training; *p* = 0.2). In D-RT, no change in epinephrine concentrations was evident from pre to postexercise (week 12 of training; *p* = 0.7); however, epinephrine concentrations were lower overall on day 2 compared to day 1 (week 12 of training; *p* = 0.02). In D-ART, when RT occurred the day before AT_high_ (week 11 of training), epinephrine concentrations were reduced overall during AT_high_ (*p* = 0.007). In D-ART, no change in epinephrine occurred from pre to postexercise at both week 11 (*p* = 0.5) and week 12 (*p* = 0.2) of training.

## 4. Discussion

Stringent management of blood glucose concentrations through intensive insulin therapy is the primary treatment strategy in order to limit the progression of CVD in patients with T1DM [2]. Indeed, D-IIT resulted in greater recovery from an I-R injury than D-CIT, supporting the deleterious effects of chronic hyperglycemia on the macrovasculature in individuals with T1DM [12, 25]. In a previous study, we reported that six weeks of high-intensity aerobic exercise led to significant improvements in I-R functional recovery [4]. Here, we demonstrate that this modality of exercise when combined with CIT can lead to comparable recovery from an I-R injury as IIT alone. It is important to note that while exercised animals were maintained in a chronic hyperglycemic state, AT_high_ exhibited similar serum fructosamine concentrations as IIT. It is likely that glycemic control played a significant role in contributing to the increased cardioprotection of IIT and AT_high_, since both exhibited similar serum fructosamine concentrations. Indeed, the negative correlation between serum fructosamine, indicative of glycemic control, and LVDP would support this finding.

While it is well-recognized that regular exercise can improve glycemic control (lowered HbA_1C_) in type 2 diabetes, results in T1DM have generally failed to show this glycemic benefit [3]. A number of factors may contribute to this lack of evidence in previous studies, including the predominant use of adolescent subjects, the use of questionnaires to estimate activity levels, or the increased food consumption that is typically associated with the initiation of an exercise program [3]. Although comparable to HbA_1c_, the measure of glycosylated hemoglobin, fructosamine is a measure of the amount of serum proteins that have undergone glycation and is thus a better marker for shorter-term glycemic control (approximately two weeks). While there is a shortage of evidence supporting increased glycemic control in T1DM following aerobic exercise [3], exercise intensity appears to play a significant role as to whether glycemic benefits are obtained [3, 26]. In the present study, the aerobic exercise training program was intensive, representing approximately 70–80% of the rats VO_2max_ [27]. The potential ability of RT to improve glycemic control (determined by HbA_1C_) in populations with T1DM is inconclusive [28], and the present results would support work citing that it has no benefit on long-term glycemia [29]. There was no improvement in fructosamine levels in D-ART, despite supplementing RT with AT_high_, suggesting that the frequency of AT_high_ may be an important factor to experience glycemic benefits. However, since food consumption was not directly measured in the current study, it cannot be completely discounted that the diets of experimental groups may not have been isocaloric.

In a previous report, we demonstrated that six weeks of RT provided little protection against an I-R injury in T1DM rats [4]. The current study demonstrated that longer term RT, conducted alone or paired with AT_high_ (D-ART), is necessary in order to provide similar levels of cardioprotection as performing strictly AT_high_. Indeed, it has been demonstrated in non-T1DM rats that short-term RT provides little cardioprotection [30]; however, if the RT is prolonged, the cardioprotective effects of this form of exercise become evident, as demonstrated by reduced infarct size following an I-R injury [31]. Again, it is of importance to note that we see unique advantages associated with RT that were not evident in other modalities of exercise. The maximal rate of pressure development (+dp/dt) and relaxation (−dp/dt) in T1DM rats were significantly improved in experimental groups utilizing RT that were not evident in D-AT_high_. It has been reported that just a single bout of RT can improve the rate of left ventricular systolic pressure in hypertensive rats undergoing Langendorf perfusion [32]. Further, Melo et al. [33] reported faster cardiomyocyte contraction and relaxation in rats following eight weeks of RT, believed to be due to increased sarcoplasmic reticulum Ca^2+^-ATPase (SERCA2a) expression. While neither +dp/dt nor –dp/dt were altered in D-AT_high_, these results may not be surprising as little change in Ca^2+^ regulatory mechanisms is reported elsewhere in rat hearts following 12 weeks of treadmill training [34]. Thus, these findings may support the incorporation of RT into the treatment of T1DM, since slowed Ca^2+^ clearing and abnormal cardiomyocyte excitation-contraction coupling are prominent in T1DM [35].

The finding that rates of pressure development and relaxation were increased in D-RT and D-ART despite no improvement in glycemia (fructosamine) indicates that other factors may contribute to changes in rates of pressure development. For example, cardiomyocytes from insulin-resistant rats have demonstrated mechanical defects and impaired Ca^2+^ handling [36, 37]. In the present investigation, we report a negative correlation between the degree of insulin resistance and the rate of developed pressure. Indeed, the experimental groups that demonstrated the greatest insulin sensitivity, D-RT and D-ART, also displayed the quickest rates of pressure development and relaxation. In the insulin-resistant state, impaired SERCA activity is well documented to contribute to cardiomyocyte dysfunction [38], and RT itself has been shown to increase SERCA expression [33]. In the present study, SERCA2 expression was not changed as a result of RT or ART. This lack of change may not reflect changes in the activity levels of this enzyme, as impaired SERCA activity has been reported in insulin-resistant animals despite normal protein content [38]. Nonetheless, the implications of insulin resistance in the recovery from an I-R injury are significant and require further investigation, given the emerging evidence of “double diabetes,” a separate classification of patients with T1DM that exhibit both insulin deficiency and resistance [39].

In seeking to explain the mechanistic means by which a specific exercise training regime may prove to be more beneficial for the functional recovery of the heart during an I-R injury, we examined cardiac Hsp70 protein expression in each of the groups [40, 41]. We observed an increase in left ventricular Hsp70 content in D-AT_high_ compared to both sedentary T1DM groups (D-CIT and D-IIT). Our laboratory, as well as others, has established the importance of exercise-induced Hsp70 expression in recovery from an I-R injury [40, 41]. This finding is in line with previous work from our laboratory that demonstrated both short- and long-term aerobic exercise can result in increased Hsp70 in the hearts of insulin-treated T1DM rats [4, 5]. Further, we showed that D-AT_high_ had higher Hsp70 expression than D-RT which supports an earlier finding by our laboratory [4]. While it is not clear why differences in the expression of Hsp70 exist between exercise modalities, it may be reflective of frequency, duration, and/or intensity of the exercise. We have previously shown that antioxidant enzymes are elevated in the myocardium following AT_high_, but not following RT [4]. Increases in myocardial antioxidant defenses have been shown to be dependent on the duration and frequency of training [42], while exercise-induced elevations in Hsp70 are known to be intensity-dependent [43]. It is plausible that D-RT did not undergo the same quantity or intensity of exercise as was achieved in D-AT_high_.

The largest barrier to exercise prescription for individuals with T1DM is exercise-induced hypoglycemia [6]. Thus, independent of which exercise provides the largest cardiovascular benefit, the risk of exercise-induced hypoglycemia must also be considered. Similar to past findings [4], D-AT_high_ resulted in a significant drop in blood glucose immediately following exercise, while D-RT did not. Interestingly, the integration of RT and AT_high_ (D-ART) did not alter the abrupt drop in blood glucose in response to AT_high_. Recently, our group has demonstrated that both sedentary and aerobically trained T1DM rats using CIT demonstrate hepatic glycogen deficiencies [15], similar to what has been reported using clinical populations [16]. Despite increased glycemic control in IIT, there was no difference in hepatic glycogen content between D-IIT and D-CIT. Further, exercise-trained T1DM rats, regardless of training modality, demonstrated significantly lower liver glycogen content. It is expected that the amount of insulin in circulation in the treatment groups contributed to different hepatic glycogen levels. Both D-CIT and D-IIT had similar exogenous insulin concentrations, which would in turn regulate glycogen storage by increasing the activity of glycogen synthase [44]. Moreover, independent of exercise modality, trained T1DM rats displayed the smallest amounts of hepatic glycogen, concurrent with the lowest exogenous insulin requirements. Despite the apparent cardiovascular benefits associated with regular exercise, the decreased hepatic glycogen content in trained T1DM rats could have implications for combatting hypoglycemia, since hepatic glycogen is a prominent source of blood glucose during glucose-demanding states [17, 18]. However, it is important to note that T1DM rats in each of the different training modalities failed to reach hypoglycemic blood glucose concentrations (less than 3 mmol/L).

In conclusion, the first objective of the present investigation was to determine if AT_high_ coupled with CIT resulted in larger cardioprotective benefits than IIT alone. Findings presented here demonstrate that when CIT was paired with AT_high_, the increase in cardioprotection from an I-R injury was similar to that of D-IIT. In fact, the current findings may suggest that CIT with AT_high_ may lead to a larger cardiac improvement in T1DM rats than IIT alone, given the potential role of elevated expression of left ventricular Hsp70 with this form of exercise. For the second objective, we determined that following long-term exercise training, both D-ART and D-RT resulted in similar levels of overall cardioprotection as D-AT_high_; although each exercise training modality did appear to provide unique benefits. For example, improved glycemic control was only evident in D-AT_high_, while the largest improvements in insulin sensitivity measures were evident in exercises that utilize resistance exercise (D-ART, D-RT). This study underlines the need to consider other factors besides glycemic control (i.e., insulin resistance) when tailoring an exercise treatment program for the patient with T1DM to reduce the risk of developing CVD.

## Figures and Tables

**Table 1:** General animal characteristics at the completion of the study.

	D-CIT	D-IIT	D-AT_high_	D-RT	D-ART
Body mass (g)	567 ± 20	598 ± 21^3,4,5^	510 ± 15	520 ± 21	534 ± 19
Blood glucose conc. (mmol/L)	15.0 ± 1.2	10.9 ± 1.2^1,3,5^	15.6 ± 0.5	12.4 ± 1.9^5^	16.7 ± 1.4
Fructosamine conc. (mmol/L)	3.0 ± 0.5	1.0 ± 0.2^1,5^	1.3 ± 0.3^1,5^	2.0 ± 0.1	2.6 ± 0.7
Exogenous insulin (IU)	27.3 ± 5.4	35.8 ± 7.8	19.0 ± 7.7	11.1 ± 7.1^2^	4.1 ± 2.0^1,2^
Insulin resistance (AU)	10,665 ± 2078^5^	16,055 ± 4558^3,4,5^	4722 ± 1988	1438 ± 62^3^	1260 ± 601^3^

Data are means ± SE. ^1^Different from D-CIT; ^2^different from D-IIT; ^3^different from D-AT_high_; ^4^different from D-RT; ^5^different from D-ART.

**Table 2:** Correlation of left ventricle mechanical performance on glycemia and insulin resistance.

	Versus fructosamine (mmol/L)		Versus insulin resistance (AU)	
	*p* value	*r*	*p* value	*r*
LVDP (AUC)	*0.01* ^∗^	−0.4	0.7	—
LVEDP (AUC)	0.8	—	0.7	—
+dp/dt (AUC)	0.7	—	*0.03* ^∗^	−0.4
−dp/dt (AUC)	0.8	—	0.5	—

^∗^Significant (*p* < 0.05).

**Table 3:** Blood glucose concentrations in response to exercise at week 11 or week 12 of training.

	Day 1		Day 2	
	Preexercise (mmol/L)	Postexercise (mmol/L)	Preexercise (mmol/L)	Postexercise (mmol/L)
D-AT_high_	15.0 ± 0.4	8.0 ± 1.1^∗^	14.6 ± 0.5	6.9 ± 1.0^∗^
D-RT	12.2 ± 1.6	12.0 ± 0.9	13.6 ± 2.2	15.1 ± 1.6
D-ART (week 11; RT then AT_high_)	14.9 ± 1.6	15.6 ± 1.2	15.4 ± 1.7	8.0 ± 1.8^∗^
D-ART (week 12; AT_high_ then RT)	16.7 ± 1.4	8.8 ± 1.4^∗^	15.2 ± 2.0	15.6 ± 1.2

Data are means ± SE. ^∗^Significantly lower than preexercise (*p* < 0.05).

**Table 4:** Epinephrine concentrations in response to exercise at week 11 or week 12 of training.

	Day 1		Day 2	
	Preexercise (pg/mL)	Postexercise (pg/mL)	Preexercise (pg/mL)	Postexercise (pg/mL)
D‐AT_high_	254.3 ± 44.6	93.1 ± 24.9	237.0 ± 43.8	298.0 ± 109.1
D-RT	320.8 ± 72.2	238.3 ± 58.2	110.0 ± 46.1^∗^	136.3 ± 66.3^∗^
D-ART (week 11; RT then AT_high_)	254.3 ± 107.2	150.8 ± 98.0	38.7 ± 9.3^∗^	57.8 ± 15.1^∗^
D-ART (week 12; AT_high_ then RT)	331.0 ± 169.8	112.6 ± 45.7	202.2 ± 33.0	184.6 ± 41.8

Data are means ± SE. ^∗^Significantly lower than day 1 (*p* < 0.05).

## Data Availability

The data used to support the findings of this study are available from the corresponding author upon request.
